# THE FORTITUDE FACTOR FOR CAREGIVERS: UNDERSTANDING THE EFFECT OF EMPLOYMENT AND CAREGIVING ON CAREGIVER WELLBEING

**DOI:** 10.1093/geroni/igae098.3900

**Published:** 2024-12-31

**Authors:** Sarah Clem, Joan Davitt, Stephanie McCorvey, Todd Becker, Jennifer Eastman, Greg Sesek

**Affiliations:** University of Maryland Baltimore, Baltimore, Maryland, United States; University of Maryland Baltimore, Baltimore, Maryland, United States; University of Maryland Baltimore, Baltimore, Maryland, United States; Washington University School of Medicine in St. Louis, St. Louis, Missouri, United States; Maryland Department of Disabilities, Maryland Department of Disabilities, Maryland, United States; Department of Human Services, Baltimore, Maryland, United States

## Abstract

Shifting support services from community-based facilities to home-based settings has highlighted the dual roles of family/friend caregivers. This study investigated challenges experienced by caregivers of vulnerable adults in one east coast state. A convenience sample (N= 2,129) of caregivers, recruited through state and local agencies, completed an anonymous online survey to enhance understanding of caregiving experiences, challenges, and service utilization patterns. This paper focused on employed caregivers who reported greater service utilization than caregivers who were not employed. Within employed caregivers, we found they made workplace compromises to balance caregiving responsibilities including: going in late, taking a less demanding job, taking a leave of absence, and going from full to part-time work. There were positive associations between the number of compromises made in the workplace with caregiver-reported stress levels, financial strain, and service utilization. To address these challenges, employed caregivers reported using various coping strategies including exercise, mental health support, spiritual practice, hobbies, and support groups. Employer support to caregivers included: flexible hours, remote work options, paid sick days, paid family leave, referral/counseling supports, and unpaid family leave. Mixed results were found for relationships between employer supports and caregiver compromises, stress levels and financial strain. Research shows that multiple demands on employees can reduce worker productivity and efficiency. Responding to trends indicating an increase of informal caregivers in the workforce, this study highlights the needed support to promote caregiver resilience and their ability to remain employed. Implications for employers, community agencies, and government systems will be discussed.

